# Gender differences in health-related quality of life measured by the Sarcoidosis Health Questionnaire

**DOI:** 10.1038/s41598-021-89383-1

**Published:** 2021-05-13

**Authors:** Łukasz Gwadera, Adam J. Białas, Witold Górski, Mikołaj A. Iwański, Joanna Miłkowska-Dymanowska, Paweł Górski, Wojciech J. Piotrowski

**Affiliations:** 1 Department of Pneumology and Allergy, World Association for Sarcoidosis and Other Granulomatous Disorders (WASOG) Recognized Sarcoidosis Clinic, Medical University of Lodz, Kopcińskiego 22, 90-153 Lodz, Poland; 2 Department of Pathobiology of Respiratory Diseases, Medical University of Lodz, Lodz, Poland

**Keywords:** Diseases, Signs and symptoms

## Abstract

Sarcoidosis is granulomatous disease, which complex etiology is yet to be fully discovered. In the majority of cases its course is self-limiting. However it can have different clinical manifestations and can be debilitating condition with great impact on health-related quality of life (HRQL). The aim of our study was to assess if there are any differences in HRQL dependent to gender. We examined a group of 33 males and 42 females (with no differences in mean age, disease activity, TLCO, FEV_1_, FVC, FEV_1_/FVC) with a use of Sarcoidosis Health Questionnaire. We revealed lower total and daily functioning score in female group. Further analyses stratified by sex and activity of the disease presented many significant differences between the groups, revealing important issues for the discussion about gender specific differences in the HRQL of patients with sarcoidosis. In spite of clinical presentation may be similar, expectations and main concerns of sarcoidosis patient can vary between females and males. Therefore, it appears that in terms of education and symptomatic treatment accents should be put differently depending on the gender of the patient. Our results may also point to a need for more gender-oriented patient-physician communication which could enable better understanding, potentially improve adherence to therapy and decrease the risk of possible complications.

## Introduction

Sarcoidosis is a systemic inflammatory disease of unknown etiology, involving formation of non-caseating granulomas in various organs. In over 90% of patients intrathoracic lymph nodes and/or lung parenchyma are involved^1^.

Health-related quality of life (HRQL) has become a crucial element of evaluation in modern patient-oriented approach to decision making in medicine. This model is especially important when treating patients with sarcoidosis as the disease itself is rarely life-threatening, and the physiological impairment is usually not severe. Therefore, each decision with regards to treatment should be considered in the context of balance between medicinal benefits and quality of life. Further, HRQL assessments facilitate patient-physician communication^2^. This is important because it serves to improve understanding about the patient’s perception of how disease and treatment affect their overall satisfaction with life in various different areas of functioning^3^.

Although gender affects epidemiology, pathophysiology, manifestation and treatment of a broad range of diseases, gender-specific oriented healthcare approach seems to still be neglected. Sarcoidosis is one of the diseases where gender plays an important role. Women more frequently suffer from sarcoidosis^4,5^, but interestingly, also tend to present less advanced radiographic stages^6^. There are also differences in organ involvement—women more frequently suffer from skin, eye, liver and peripheral lymph node involvement^6–8^. Also, manifestations of Löfgren's syndrome differ between men and women, with erythema nodosum found more often in women, whereas a marked periarticular inflammation of the ankles or ankle arthritis without erythema nodosum is seen primarily in men^7^.

Two genders manifest significant biological, behavioral, social and cultural differences. Sex differences are also visibly reflected in social conditions, lifestyles, health perception, and health care behaviors^9^. Therefore, it is not surprising that gender, among other various factors, significantly influences HRQL. However, despite increasing evidence that points to the importance in the understanding of this field, knowledge about gender differences specific to HRQL in sarcoidosis patients is still relatively scarce.

The purpose of our study was to identify associations between gender and HRQL scores using the Sarcoidosis Health Questionnaire (SHQ). The target of our study was not only limited to the three main domains described by this tool (daily functioning, physical and emotional functioning), but also focused on the results of individual questions which, when analyzed separately, may lead to important clinical conclusions.

## Patients and methods

This study is a single institution, prospective analysis of patients admitted to the Department of Pneumology and Allergy of the Medical University of Lodz or the adjacent Outpatient Clinic. The study procedures were approved by the Ethical Committee for Human Studies of the Medical University of Lodz. All study participants gave informed written consent to participate in the study. The study was performed in accordance with the Declaration of Helsinki.

### Inclusion and exclusion criteria

All study participants were non-smoking, adult Caucasians with a confirmed (in the past as well as newly made) diagnosis of sarcoidosis. The diagnosis was based on histopathological examination or on typical clinical and radiological findings—Lofgren’s syndrome, chest CT suggestive of sarcoidosis supported by additional test results (bronchoscopy, laboratory test) after exclusion of other conditions. To avoid potential skewed results, we did not enroll patients dealing with any significant stressful life situation that was not related to their health status (e.g. divorce, loss of relative, unemployment, clinically proven depression, etc.).

We excluded patients with any other pulmonary or autoimmune disease as well as patients with confirmed extrapulmonary sarcoidosis of the heart, central nervous system, skin and/or eyes. Other form of extrapulmonary sarcoidosis (e.g.: peripheral lymphadenopathy, liver sarcoidosis) did not exclude the patients. We also excluded patients with acute coronary syndrome, severe heart failure (NYHA III and more), chronic kidney disease, any previously diagnosed mental illness and/or a history of malignancy in their past medical history.

As a potentially significant cofounding factor, we also excluded patients currently receiving oral steroids or other medications for sarcoidosis other than non-steroid anti-inflammatory drugs and other painkillers.

Patients were divided into study subgroups according to gender and disease activity, into active and inactive sarcoidosis group. Inactive sarcoidosis was diagnosed when complete radiological and clinical resolution was achieved or when radiological stabilization was noted in a patient without other signs or symptoms of pulmonary or extrapulmonary disease. Active sarcoidosis was diagnosed when one of the following criteria was met: present symptoms of pulmonary sarcoidosis (e.g. cough, dyspnoea), Loefgren syndrome and up to 3 month after, signs of extrathoracic sarcoidosis, radiological progression or worsening of the lung function tests, increased percentage of lymphocyte in broncho-alveolar lavage fluid, hypercalcemia or hypercalciuria.

### HRQL

To measure HRQL we used the validated Polish version of the SHQ^10^. This is a short, self-administered questionnaire for persons with sarcoidosis of varying disease burden and organ involvement^11^. SHQ is a comprehensive measure analyzing three domains: daily functioning, physical functioning, and emotional functioning. Each domain is assessed by questions related to its characteristics, scored from 1 to 7. Then, scores were calculated according to the equation presented by the authors of the questionnaire^11^.

### Statistical analysis

Continuous data were presented as mean with standard deviation (SD) or median with interquartile range (IQR) from LQ (25%) to UQ (75%), depending on the data normality. Variables were compared using the unpaired Student’s t test, Welch t test or the Wilcoxon rank sum test with continuity correction, depending on data normality and homogeneity of variance. The correlation analysis was performed using Spearman’s test.

Categorical data were presented as absolute value and percentage. Such data were compared using Pearson’s Chi-square test (with Yates correction if appropriate) or Fisher’s exact test concordantly with test assumptions.

Analysis was performed using R software (R: A language and environment for statistical computing, R Core Team, R Foundation for Statistical Computing, Vienna, Austria, 2018, https://www.R-project.org).

## Results

### Study characteristics

We enrolled 75 patients—42 (56%) females and 33 (44%) males. Characteristics of study participants are presented in Table 1.

**Table 1:** Characteristics of study participants.

Characteristic	Result
Age, year, median (IQR)	43 (39–54)
**Organ systems involved, n (%)**	
Lungs	71 (94.67)
Lymph nodes	40 (53.33)
**Sarcoidosis activity, n (%)**	
Active	43 (57.33)
Non-active	32 (42.67)
Active Löfgren’s syndrome	12 (16)
**Comorbidities, n (%)**	
Arterial hypertension	7 (9.33)
GERD	2 (2.67)
Rhinosinusitis	1 (1.33)
Cardiac arrhythmia^a^	1 (1.33)
Hypothyroidism^b^	3 (4)
FEV_1_, %, median (IQR)	90.12 (84.53–94.73)
FVC, %, median (IQR)	93 (86.13–97)
FEV_1_/FVC, median (IQR)	0.78 (0.76–0.8)
T_L,CO_, %, median (IQR)	94 (89–98)

^a^Other than atrial fibrillation, not related to sarcoidosis heart involvement; ^b^currently in euthyreosis.

Female group and male group did not differ according to age (*P* = 0.14), sarcoidosis activity (*P* = 0.33), and presence of active Löfgren’s syndrome (*P* = 0.86). There were no between gender differences according to pulmonary function test results: T_L,CO_ (*P* = 0.86), FEV_1_ (*P* = 0.48), FVC (*P* = 0.96), and FEV_1_/FVC (*P* = 0.95).

We found a significantly lower total SHQ score in the female group—4.64 vs 6.14 (*P* = 0.02). Analysis of individual domains revealed a significant difference only in physical functioning: 4.28 vs 4.87 (*P* = 0.02). However, in detailed analyses we found significant differences in questions from all domains as follows: “felt bodily pain” (*P* = 0.006), “bothered by sarcoidosis skin or hair problems” (*P* = 0.0005), “bothered by eye or eyesight problems” (*P* = 0.02), “had joint pains” (*P* = 0.02), “been bothered by headaches” (*P* = 0.0008), “worried about the amount of pain and discomfort” (*P* = 0.047) and “felt satisfied with the appearance of your body” (*P* = 0.049). All results of detailed analysis are presented in Table 2.

**Table 2:** Sarcoidosis health questionnaire items by domain, stratified by gender.

Domain and questions	Males n = 33 (44%)	Females n = 42 (56%)	Total n = 75	P value
**Daily functioning**	**4.65 (1.02)**	**4.15 (1.11)**	**4.37 (1.09)**	**0.05**^**$**^
Felt full of energy	4 (3–5)	4 (2–5)	4 (2.5–5)	0.67^#^
*Felt bodily pain*	**6 (4–6)**	**4 (3–5)**	**4 (3–6)**	**0.006**^**#**^
Felt that you need medication to function on a daily basis	6 (4–7)	4.5 (3–6)	5 (3–6.5)	0.1^#^
Felt confident in yourself and your abilities	5 (4–6)	5 (4–5)	5 (4–5)	0.44^#^
Felt that you accomplished all that you wanted	4 (3–5)	4 (3.25–5)	4 (3–5)	0.82^#^
Felt that everything you did took a lot of effort	4 (3–6)	3.5 (3–4)	4 (3–5)	0.07^#^
Felt discouraged by recent weight gain	6 (4–6)	5 (3–5.75)	5 (3–6)	0.06^#^
Felt sarcoidosis controls your life	4 (3–5)	4 (2.25–5)	4 (3–5)	0.88^#^
Had a good night’s sleep	4.5 (3–6)	4 (3–5)	4 (3–5)	0.4^#^
*Bothered by sarcoidosis skin or hair problems*	**7 (6–7)**	**4 (3–6)**	**6 (4–7)**	**0.0005**^**#**^
Felt that you were as healthy as others your age	4 (3–5)	4 (3–5)	4 (3–5)	0.67^#^
Felt that your physical problems interfered with your social activities when with family and friends	4 (4–6)	4 (3–5)	4 (3.5–5.5)	0.48^#^
*Bothered by eye or vision problems*	**6 (4–7)**	**4 (4–6)**	**5 (4–6)**	**0.02**^**#**^
**Physical Functioning**	**4.87 (1.2)**	**4.28 (0.89)**	**4.54 (1.07)**	0.02*
Felt your breathing was completely comfortable during normal daily activities	4 (3–6)	4 (3–5.75)	4 (3–6)	0.37^#^
Felt shortness of breath walking upstairs, the length of a city block, or up a small hill	4 (2–6)	4 (3–5)	4 (2.5–5)	0.67^#^
*Had joint pain*	**4 (4–7)**	**4 (2.25–4.25)**	4 (3–6)	**0.02**^**#**^
Had a cough	5 (3–7)	4 (3.25–5.75)	5 (3–6)	0.22^#^
*Been bothered by headaches*	**6 (4–6)**	**4 (3.25–5)**	5 (4–6)	**0.0008**^**#**^
Experienced wheezing	6 (5–7)	5 (4–7)	6 (4–7)	0.32^#^
**Emotional functioning**	**4.77 (0.99)**	**4.42 (1.1)**	**4.58 (1.06)**	**0.16**^**$**^
Worried sarcoidosis might flare up or worsen	4 (3–6)	4 (2.25–5)	4 (2.5–5)	0.54^#^
Expect your health to be good in the future	5 (4–6)	5 (4–6)	5 (4–6)	0.46^#^
Satisfied with the support of family and friends	5 (4–6)	5 (4–6)	5 (4–6)	0.53^#^
*Worried about the amount of pain and discomfort*	**5 (4–6)**	**4 (3–5)**	**5 (4–6)**	**0.047**^**#**^
Felt mood swings	4 (4–5)	4 (3.25–5)	4 (4–5)	0.49^#^
Felt depressed	6 (5–7)	5 (4–6.75)	6 (4–7)	0.27^#^
*Felt satisfied with the appearance of your body*	**5 (4–6)**	**4 (3–5)**	**4 (3.5–5)**	**0.049**^**#**^
Felt that you could concentrate easily	5 (4–6)	4.5 (4–6)	5 (4–6)	0.81^#^
Been discouraged by physical limitations in performing your normal daily activities or your job	4 (3–6)	4 (2.25–4.75)	4 (3–5)	0.17^#^
Felt that your emotional problems affected your relationships with family, friends, or coworkers	5 (4–6)	5 (3–6)	5 (3.5–6)	0.19^#^
**Total**	**6.14 (4.27–12.89)**	**4.64 (4.11–6.11)**	**5.34 (4.2–8.95)**	**0.02**^**#**^

Data are presented as mean (SD) or median (IQR). Presented P values are for: ^#^Wilcoxon rank sum test, ^$^Student or *Welch t test.

### Sarcoidosis activity

When the subgroups depending on disease activity, not gender, were analyzed, some associations with SHQ results were found. In active sarcoidosis, there were significantly lower scores for daily functioning, but not for physical functioning and total score (Table 3).

**Table 3:** Differences between active and non-active sarcoidosis.

Domain/question	Active sarcoidosis	Non-active	P value
**Daily functioning**	**4 (3.5–4.73)**	**4.8 (4–5.73)**	**0.006**
Felt you were full of energy	4 (2–4)	4 (3–6)	0.02
Felt that everything you did took a lot of effort	3 (3–4)	4 (3–6)	0.04
Felt that you accomplished all that you wanted	4 (3–5)	4.5 (4–6)	0.02
Felt bodily pain	4 (3–5.5)	5.5 (4–6)	0.03
Had a good night’s sleep	4 (2–5)	5 (3.75–6)	0.01
Felt sarcoidosis controls your life	3 (2–4)	4 (3–6)	0.01
Felt that your physical problems interfered with your social activities when with family and friends	4 (3–5)	5 (4–6)	0.001
Felt confident in yourself and your abilities	4 (4–5)	5 (4–6)	0.02
Felt you were as healthy as others your age	4 (3–4)	5 (4–6)	0.0002
**Emotional functioning**	**4.2 (3.8–5)**	**4.85 (4.18–5.83)**	**0.02**
Worried about the amount of pain and discomfort	4 (3–5)	5 (4–6)	0.04
Felt that your emotional problems affected your relationships with family, friends, or coworkers	4 (3–5)	5.5 (4–6)	0.02
Been discouraged by physical limitations in performing your normal daily activities or your job	3 (3–4)	5 (4–6)	0.001
**Physical functioning**	4.17 (3.92–4.83)	5.08 (4–5.63)	0.07
Felt your breathing was completely comfortable during your normal daily activities	4 (3–5)	5 (4–6)	0.03
Total score	5.13 (4.14–12.33)	5.19 (4.18–5.96)	0.21

Data are presented as median (IQR). Presented P values are for Wilcoxon rank sum test.

For individual questions, there were significant differences in the following questions: “Felt you were full of energy”, “Felt your breathing was completely comfortable during your normal daily activities”, “Worried about the amount of pain and discomfort”, “Felt that everything you did took a lot of effort”, “Felt that your physical problems interfered with your social activities when with family and friends”, “Felt that you accomplished all that you wanted”, “Felt bodily pain”, “Felt that your emotional problems affected your relationships with family, friends, or coworkers”, “Felt sarcoidosis controls your life”, “Had a good night’s sleep”, “Felt confidence in yourself and your abilities”, “Felt you were as healthy as others your age”, and for “Been discouraged by physical limitations in performing your normal daily activities or your job” (Table 3).

Then, we compared both sexes within active and non-active sarcoidosis group. In the female active sarcoidosis group we observed significantly lower scores for the following questions: “Been bothered by headaches”, “Bothered by sarcoidosis skin or hair problems”, “Felt discouraged by recent weight gain”, “Felt bodily pain”, “Felt satisfied with the appearance of your body”, but not for any of the main domains. Among female patients with non-active sarcoidosis, we noticed lower scores for physical functioning and for the following questions: “Been bothered by headaches”, “Felt bodily pain” and “Experienced wheezing” (Table 4).

**Table 4:** Differences between sexes in active and non-active sarcoidosis.

Domain and questions	Females with active sarcoidosis	Males with active sarcoidosis	P value
Been bothered by headaches	4 (3.25–5)	6 (4–6)	0.03
Bothered by sarcoidosis skin or hair problems	4 (3–4.75)	7 (6–7)	0.002
Felt discouraged by recent weight gain	4.5 (2.25–5)	6 (5–7)	0.01
Felt bodily pain	3.5 (3–4.75)	5 (4–6)	0.04
Felt satisfied with the appearance of your body	4 (2.25–4)	5 (4–6)	< 0.001

Domain and questions	Females with non-active sarcoidosis	Males with non-active sarcoidosis	P value
Physical functioning	4.17 (3.96–5.2)	5.83 (5.13–6.21)	0.009
Been bothered by headaches	4 (3.75–6)	6 (6–7)	0.006
Felt bodily pain	4 (3.75–6)	6 (5.75–6.25)	0.03
Experienced wheezing	5 (4–6)	6.5 (6–7)	0.004

Data are presented as median (IQR). Presented P values are for Wilcoxon rank sum test.

We made also similar analysis for the genders separately, noticing that the active period of sarcoidosis influences SHQ in men and in women in different aspects. Namely, female patients with active disease had a lower score for daily functioning and a lower score in the following questions: “Bothered by sarcoidosis skin or hair problems”, “Worried about the amount of pain and discomfort”, “Felt that your physical problems interfered with your social activities when with family and friends”, “Felt that your emotional problems affected your relationships with family, friends, or coworkers”, “Felt satisfied with the appearance of your body”, “Felt you were as healthy as others your age” and “Been discouraged by physical limitations in performing your normal daily activities or your job” (Table 5).

**Table 5:** Sex differences in active sarcoidosis.

Domain and questions	Females with non-active sarcoidosis	Females with active sarcoidosis	P value
Daily functioning	4.31 (3.88–5.63)	3.96 (3.4–4.48)	0.04
Bothered by sarcoidosis skin or hair problems	6 (4–6.25)	4 (3–4.75)	0.022
Worried about the amount of pain and discomfort	5 (4–6)	4 (3–4.75)	0.04
Felt that your physical problems interfered with your social activities when with family and friends	5 (4–6.25)	4 (2.25–4)	0.005
Felt that your emotional problems affected your relationships with family, friends, or coworkers	5 (4–6)	4 (3–5)	0.04
Felt satisfied with the appearance of your body	5 (3–6)	4 (2.25–4)	0.03
Felt you were as healthy as others your age	5 (4–6)	3.5 (3–4)	0.006
Been discouraged by physical limitations in performing your normal daily activities or your job	4.5 (4–6)	3 (2–4)	0.003

Domain and questions	Males with non-active sarcoidosis	Males with active sarcoidosis	P value
Daily functioning	5.38 (4.46–5.94)	4.15 (3.62–5.23)	0.03
Physical functioning	5.83 (5.13–6.21)	4.17 (4–5)	0.01
Felt your breathing was completely comfortable during your normal daily activities	6 (4.75–7)	4 (3–5)	0.03
Felt shortness of breath walking upstairs, the length of a city block, or up a small hill	5.5 (3.75–7)	3 (2–4)	0.04
Had a good night’s sleep	6 (4.75–6)	4 (3–5)	0.02
Experienced wheezing	6.5 (6–7)	5 (4–7)	0.02
Felt confidence in yourself and your abilities	5.5 (4.75–6)	4 (4–5)	0.04
Felt you were as healthy as others your age	5 (4–6)	4 (3–5)	0.009

Data are presented as median (IQR). Presented P values are for Wilcoxon rank sum test.

On the other hand, male patients with active disease had lower scores for daily and physical functioning. When particular questions were analyzed, differences were found in following: “Felt your breathing was completely comfortable during your normal daily activities”, “Felt shortness of breath walking upstairs, the length of a city block, or up a small hill”, “Had a good night’s sleep”, “Experienced wheezing”, “Felt confidence in yourself and your abilities”, and “Felt you were as healthy as others your age” (Table 5).

## Discussion

The results of our study showed a significantly decreased HRQL in the female group of patients. In a more detailed analysis, the only domain which was statistically significantly decreased was physical functioning. This result is concordant with a previous study by Bourbonnais et al.^12^. Authors did not analyze individual elements of the questionnaire, but instead, analyzed pulmonary function tests and six-minute walking test results. After this analysis, they suggested that the following factors were associated with poor HRQL differences based on gender: reduction in T_L,CO_, reduction in 6MWT distance, and an increased sensation of dyspnea. Our groups did not differ significantly with regards to pulmonary function tests.

After stratification by sarcoidosis activity, we noticed that in the active sarcoidosis group, the sexes did not differ according to any main domains, but females had significantly lower scores concerning headaches, skin or hair problems, gaining weight, body appearance and bodily pain. However, in non-active sarcoidosis group the differences in the physical functioning appeared—women had significantly lower score in this domain. Similarly to the active sarcoidosis group, females had lower scores for questions concerning headaches and bodily pain when compared to males. Additionally, significant difference in wheezing experience between women and men groups with non-active sarcoidosis could be found. This results (except of bodily pain) present different constellation of answers than baseline stratification by activity within whole study group, revealing new areas for further research and improving communication with patients in everyday clinical practice. We also made similar analysis from another point of view—presenting sexes separately and comparing active and non-active sarcoidosis within these groups. We noticed dissimilar pattern of differences, which is supplementary to previous analysis. Namely, among female patients those with active disease had a lower score for daily functioning than those with non-active. In terms of the questions related to physical appearance, social functioning, and emotional state, women with active sarcoidosis were more *worried about the amount of pain and discomfort* and were more *discouraged by physical limitations in performing their normal daily activities or job*. On the other hand, active disease in male patients affected daily functioning as well as physical functioning domain. In particular questions analysis, we observed different pattern of answers. Notably, significant differences were observed in questions related mainly to somatic health (i.e. physical activity, symptoms) and self-confidence. When we analyze Table 5 in the context of the baseline comparison of active and non-active sarcoidosis (regardless the sex), we can notice that activity of sarcoidosis affects the quality of life in different way in women and men. Differences in scores in daily functioning domain as a whole and in “Felt you were as healthy as others your age” question were the only common for females, males and the general group. This additional comparison presents stratification according to activity in different context. In our opinion, these two comparisons, analyzed in the light of the baseline activity comparison, present this issue in broader, richer context.

After extensive review of the literature of the field, we did not identify a similar study which used our approach and analysis of individual elements of the questionnaire. This is a novel contribution to this field that complements existing knowledge by indicating areas for gender-oriented improvement in the understanding of patient’s perceptions, especially fears about the disease symptoms and prognosis. As a result, this would better facilitate patient-physician communication and, potentially, decrease the level of anxiety, further improving adherence to therapy.

Our results can be divided into patients with pattern of answers associated more with pain and discomfort (feeling of bodily or joint pain, headaches, worries about the amount of pain and discomfort) and those bothered by physical appearance (skin/hair problems), eye or vision problems.

There is a strong body of evidence which documents male and female gender discrepancies in perception of pain^13,14,14–16^. This includes studies that evidently show a female predisposition to migraine and medication-overuse headache^17,18^, justifying our observations. However, despite a clear epidemiological and psychological association, the results indicate the need for an individualized approach to pain treatment and a need to identify the specific needs of female patients in this area. We should also be aware of the risk of medication-overuse in this group and thus, pay more attention towards, firstly, potential complications of NSAID overuse, and secondly, directed at the prevention of this phenomenon.

Information gathered involving eye and vision problems was difficult to analyze because none of the patients in this study experienced sarcoidosis-related visual symptoms. However, there is a wide range of information on these topics that is readily and publicly available. This information may raise anxiety among patients. As a result, it is important to maintain open channels of communication with the patient regarding issues such as this in order to calm patient’s worries by means of increased education and, in this particular case, regular ophthalmological assessment.

Differences in scores in the question “bothered by sarcoidosis skin or hair problems” would be justified by presence of Löfgren’s syndrome. According to literature of the field, it is important to highlight that females present with different manifestations of Löfgren's syndrome, predominantly with erythema nodosum, while in men more common presentation is periarticular inflammation of the ankles or ankle arthritis without erythema nodosum^7^. Therefore, erythema nodosum, affecting esthetical self-esteem, may influence this result markedly. In our study, the number of patients presenting with Löfgren’s syndrome is too low, precluding reliable analysis. However, presented issue indicates that this topic also requires further research.

A relatively low number of patients and a single-center character of study are the main limitations of our research. However, the strict exclusion criteria responsible for a lower number of participants also contributed to a more homogenous study population.

## Conclusions

Women with sarcoidosis have a lower HRQL score measured by SHQ. This group of patients presents significantly lower scores in questions that attempt to identify the degree of physical functioning impacted by disease.

Specific areas connected with lower HRQL are associated with pain and discomfort, physical appearance and eye or vision problems. These results may point to a need for more gender-oriented patient-physician communication which could enable better understanding, potentially improve adherence to therapy and decrease the risk of possible complications.

## Data Availability

The datasets used and/or analyzed during the current study are available from the corresponding author on reasonable request.
